# A combination of long- and short-read genomics reveals frequent p-arm breakpoints within chromosome 21 complex genomic rearrangements

**DOI:** 10.1016/j.gimo.2024.101863

**Published:** 2024-06-28

**Authors:** Jakob Schuy, Kristine Bilgrav Sæther, Jasmin Lisfeld, Marlene Ek, Christopher M. Grochowski, Ming Yin Lun, Alex Hastie, Susanne Rudolph, Sigrid Fuchs, Kornelia Neveling, Maja Hempel, Alexander Hoischen, Maria Pettersson, Claudia M.B. Carvalho, Jesper Eisfeldt, Anna Lindstrand

**Affiliations:** 1 Department of Molecular Medicine and Surgery and Center for Molecular Medicine, Karolinska Institutet, Stockholm, Sweden; 2 Science for Life Laboratory, Karolinska Institutet Science Park, Solna, Sweden; 3 Institute of Human Genetics, University Medical Center Hamburg-Eppendorf, Hamburg, Germany; 4 Department of Clinical Genetics and Genomics, Karolinska University Hospital, Stockholm, Sweden; 5 Department of Molecular and Human Genetics, Baylor College of Medicine, Houston, TX; 6 Pacific Northwest Research Institute, Seattle, WA; 7 Bionano Genomics, San Diego, CA; 8 Gemeinschaftspraxis für Humangenetik und Genetische Labore, Hamburg, Germany; 9 Department of Human Genetics, Research Institute for Medical Innovation, Radboud University Medical Center, Nijmegen, The Netherlands; 10 University Heidelberg, Institute of Human Genetics, Heidelberg, Germany; 11 Department of Internal Medicine, Radboud Expertise Center for Immunodeficiency and Autoinflammation and Radboud Center for Infectious Disease (RCI), Radboud University Medical Center, Nijmegen, The Netherlands; 12 Genomic Medicine Center Karolinska, Karolinska University Hospital, Stockholm, Sweden

**Keywords:** Chromosome 21, Complex genomic rearrangement, Down syndrome critical region, Long-read genome sequencing, Optical genome mapping

## Abstract

**Purpose:**

Although chromosome 21 is the smallest human chromosome, it is highly relevant in the pathogenicity of both cancer and congenital diseases, including Alzheimer disease and trisomy 21 (Down syndrome). In addition, cases with rare structural variants (SVs) of chromosome 21 have been reported. These events vary in size and include large chromosomal events, such as ring chromosomes and small partial aneuploidies. The p-arm of the acrocentric chromosome 21 was devoid of reference genomic sequence in GRCh37 and GRCh38, which hampered our ability to solve genomic rearrangements and find the mechanism of formation of disease-causing SVs. We hypothesize that conserved satellite structures and segmental duplications located on the p-arm play an important role in the formation of complex SVs involving chromosome 21.

**Methods:**

Three cases with complex chromosome 21 rearrangements were studied with a combination of short-read and long-read genome sequencing, as well as optical genome mapping. The data were aligned to the T2T-CHM13 assembly.

**Results:**

We were able to resolve all 3 complex chromosome 21 rearrangements in which 15, 8, and 26 breakpoints were identified, respectively. By comparing the identified SV breakpoints, we were able to pinpoint a region between 21p13 and 21p12 that appears to be frequently involved in chromosome 21 rearrangements. Importantly, we observed acrocentric satellite DNA at several breakpoint junctions suggesting an important role for those elements in the formation of complex SVs.

**Conclusion:**

Taken together, our results provide further insights into the architecture and underlying mechanisms of complex rearrangements on acrocentric chromosomes.

## Introduction

Chromosome 21 is the smallest human chromosome, totaling 45 Mb DNA sequence and harboring approximately 221 protein-coding genes (UCSC, T2T-CHM13v2.0), 635 noncoding genes, and 205 pseudogenes. Although the majority of the protein-coding genes are mapped to the q-arm, the p-arm of this acrocentric chromosome is rich in low-complexity sequences. These regions contain satellite DNA, retrotransposons (eg, LINEs and *Alu*) and low-copy repeats, which have been known to play a role in genomic integrity, chromosomal rearrangements and cell division.^1^^,^^2^

The small size and the limited number of genes on chromosome 21 allow trisomy 21 to be one of the viable chromosomal aneuploidies in humans and the cause of Down syndrome (DS; MIM#190685). By detailing participants with duplications on chromosome 21, having a partial trisomy for those regions, a minimal region of overlap has been identified defining critical regions and genes for specific parts of the DS phenotype.^3^ Similarly, although complete nonmosaic monosomy 21 is not compatible with life, rare cases with partial heterozygous loss of chromosome 21 segments have been reported and linked to developmental delay, craniofacial abnormalities, and malformations in the cardiovascular and/or pulmonary systems.^4^, ^5^, ^6^

Complex genomic rearrangements (CGRs), defined as more than 1 structural variant (SV) *in cis*, affecting either a single or multiple chromosomes, are more frequent disease-causing variants than previously thought.^7^ In such cases, the junctions often carry characteristic sequencing signatures that may reveal the mechanism leading to the rearrangements, e.g., nonallelic homologous recombination,^8^ microhomology-mediated break-induced replication, and canonical nonhomologous end joining each require decreasing size of shared nucleotide similarity during DNA double-strand break processing and repair.^9^

The complex challenge (ie, to detect and correctly resolve CGRs) involves sequencing the junctions at nucleotide level often in combination with conventional and molecular cytogenetic investigations such as karyotyping, fluorescence in situ hybridization (FISH) and chromosomal microarray analysis (CMA). Although paired-end short-read genome sequencing (srGS) is useful in many cases, it is often an advantage to use additional techniques such as linked-read genome sequencing (liGS) and long-read genome sequencing (lrGS) for phasing the SVs, as well as optical genome mapping (OGM) to bridge larger stretches of repetitive sequences and to detect multiple junctions in cis.^7^

The incompleteness of the available human reference genomes, GRCh37 and GRCh38,^10^^,^^11^ in certain genomic regions, such as the centromeres, telomeres, and the repetitive sequences on the p-arms of acrocentric chromosomes, render rearrangements in such regions more challenging. However, the recently released telomere-to-telomere reference (T2T-CHM13) has improved coverage within regions that have been difficult to visualize in previous genome builds, eg, p-arms of acrocentric chromosomes.^12^

In this study, we report the genomic structure of chromosome 21 derivatives detected in 3 participants. We show how T2T-CHM13, lrGS, and OGM can be used to detect hidden SVs and how it enables solving CGRs, particularly on the p-arm of chromosome 21, which has been missing from detailed characterizations of genomic rearrangements.

## Materials and Methods

### Study participants

We report genomic findings from 3 CGR participants, RD_P505, RD_P26, and RD_P01. RD_P505 was clinically investigated at the Institute of Human Genetics, University Medical Center Hamburg-Eppendorf, Hamburg, Germany. The participants RD_P26 and RD_P01 were recruited from regular clinical investigations at the Clinical Genetics Department, Karolinska University Hospital, Stockholm, Sweden. For all 3 cases parental samples were also available.

RD_P505 is a 22-year-old male, born to healthy unrelated parents, who presented with moderate developmental delay, short stature, microcephaly, and severe hearing impairment. He started walking at age 5 and displayed dysmorphic features overlapping with DS, including low-set ears, protruding right ear, facial asymmetry, small and slanting jaw, long nose, nasal voice, upslanting palpebral fissures, short fingers, and single transverse palmar crease. The results from brain magnetic resonance imaging and echocardiography were unremarkable.

The clinical presentations of RD_P26 and RD_P01 were reported previously. In brief, RD_P26, a female child born to healthy unrelated Swedish parents, presented with hemifacial microsomia and speech and developmental delay. Clinical and cytogenetic data on the CGR, a ring chromosome, were reported previously where transcriptome data from induced-pluripotent stem cells were provided.^5^

RD_P01, a female child born to healthy unrelated parents, presented at birth with developmental delay, dysmorphic features, and a heart anomaly. She passed away at 5 months of age. The *de novo* CGR originated from the paternal chromosome. The data were published previously providing the clinical and cytogenetic results^6^ and the srGS data.^13^

## Cytogenetic analysis, FISH, and CMA

RD_P505 was analyzed with karyotyping and metaphase FISH according to standard protocols. The applied FISH probes were TelVysion 21q SpectrumOrange and SpectrumGreen (*VIJyRM2029*) and LSI AML (21q22) Spectrum Aqua to label the subtelomeric region 21qter and *RUNX1* in 21q22, respectively (Abbott/Vysis).

The nucleolar organizing region (NOR) was stained with the AgNOR staining according to standard protocols.^14^^,^^15^ The 180K Array (Agilent) was used to detect CNVs by CMA.

### Short-read genome sequencing

Genomic DNA derived from peripheral blood was sequenced for srGS on Novaseq 6000 (RD_P505), Hiseq2500 (RD_P01), and Hiseq Xten (RD_P26) at National Genomics Infrastructure, Stockholm, Sweden, and analyzed according to the protocol described previously.^16^ In short, sequencing approaches harbored 2 protocols: 30X PCR-free (all participants) and 3X 2.5 kb insert size mate-pair (only RD_P26). SV calling was performed with the FindSV pipeline (https://github.com/J35P312/FindSV), which combines the bioinformatic tools CNVnator V0.3.2 and TIDDIT.^16^, ^17^, ^18^

### Linked-read genome sequencing

10X Genomics Chromium genome sequencing was used for liGS analysis on RD_P505 and RD_P26, including parents followed by phasing the parents as described.^16^ In summary, DNA samples from peripheral blood were sequenced with Illumina Hiseq Xten, passed into Supernova V2.0.0 pipeline for *de novo* assembly and alignment using Long Ranger V2.1.2, which includes SV calling for downstream analysis.

### lrGS long-read genome sequencing

All participants were sequenced using the Pacific Biosciences (PacBio) Sequel II platform. We used 1 flow cell per sample (mean coverage of 5.4 reads). The PacBio HiFi sequencing was performed at the National Genomics Infrastructure, Uppsala, using standard protocols. Alignment, as well as SV calling, was performed using LOng-read Multiomic PipelinE (https://github.com/kristinebilgrav/LOMPE), which combines minimap2, bcftools, Sniffles 1 and CNVpytor.

### Optical genome mapping

For RD_P26, ultra-high molecular weight DNA was isolated from 1.5 million cultured fibroblasts using the SP Blood and Cell Culture DNA isolation kit and provided protocol from the supplier (Bionano Genomics). For labeling 750 ng (2.65 × 10^−8^ oz) of input genomic DNA, the Direct Label and Stain DNA Labeling Kit was used, whose procedure includes enzyme DLE-1 and DL-green fluorophores for labeling, as well as a DNA backbone counterstaining. The gDNA was loaded on a Saphyr chip (G2.3) and imaged on a Saphyr instrument. The *de novo* assembly and data alignment were performed with Bionano Solve v3.6. For direct visualization of the data, Bionano Access software v1.6.1 was used. Applied Kits, read-out instruments, bioinformatic pipeline, and reference genome come from Bionano Genomics.

### Analysis of genomic data

The combined data from all sequencing platforms and OGM were aligned to the reference T2T-CHM13 from the Telomere-to-Telomere Consortium.^12^ The aligned data from all platforms were visualized in integrative genome viewer (IGV) V2.6.1 simultaneously as separate tracks.^19^ Manual inspection was utilized for the analysis of junctions, enabling us to verify breakpoints and build the derivative chromosomes by following soft-clipped read pairs. The locations of interest were provided by SV call files provided for method-specific SV callers and by blasting soft-clipped reads with the UCSC tool Basic Local Alignment Search Tool alike tool.^20^ Copy-number variants were determined by read depth of the aligned srGS data obtained using Mosdepth 0.3.3 and visualized using the latest version of VizCNV,^21^ which is publicly available at https://github.com/BCM-Lupskilab/VizCNV. The visual representation of resolved rearrangements was created using the Circos package in Perl version 5.32.0.^22^ Initially, a manually generated chromosome with G-bands served as the backbone, aligned with T2T-CHM13 coordinates. Subsequently, junction coordinates were incorporated as highlights and links in the Circos plot.

All liGS parental data were also loaded as additional tracks in IGV, allowing phasing and haplotyping of both SVs and single-nucleotide variants (SNVs). SNVs were called using GATKs best practice guideline for germline short variant discovery for SNVs and Indels using bamtools V2.5.1 (https://github.com/pezmaster31/bamtools), Picard tools V2.23.4 (http://broadinstitute.github.io/picard), GATK V4.1.4.1 and samtools V1.10.^23^^,^^24^ Phased SNVs were filtered for a score >40 before grouping into detected SVs. Finally, we calculated the ratio of maternal and paternal SNVs compared with the total number of SNVs and a 2-sided *t* test was used to check for statistical significance.

## Results

Three participants, RD_P505, RD_P26, and RD_P01, have been thoroughly investigated with cytogenetic and sequencing techniques to determine the exact breakpoint positions, as well as the length and orientation, of the duplications and deletions involved (Supplemental Table 1).

### RD_P505: Chromoanasynthesis with only duplications

**Figure 1:**
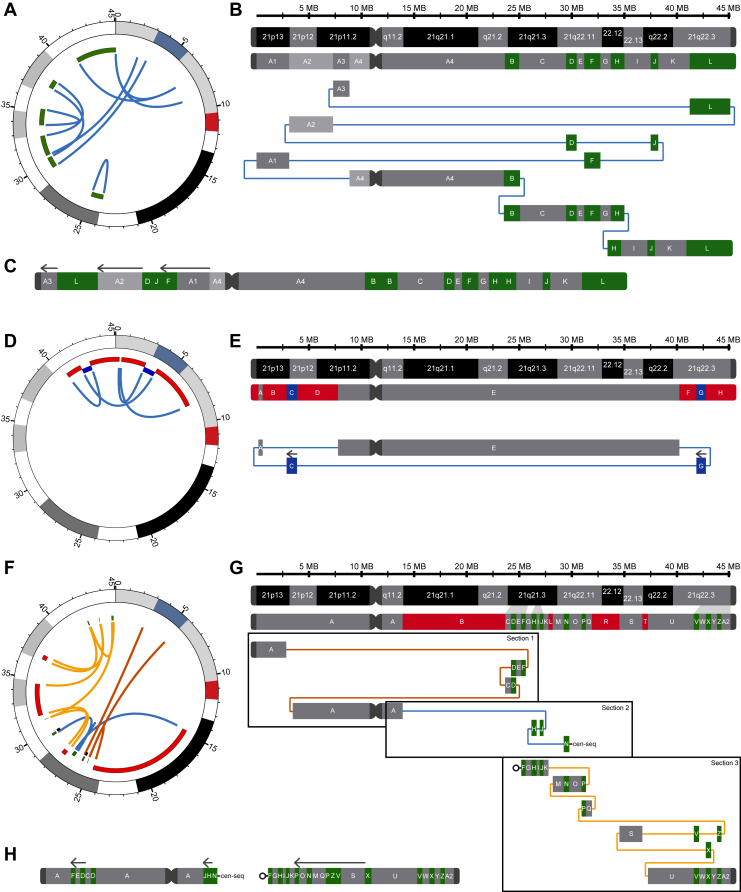
**Genomic analysis of RD_P505, RD_P26 and RD_P01.** A-C. Circos plot for RD_P505, labeled for the centromere (CEN) and G-banding (black and gray), shows all breakpoint junctions connecting the duplications (green). The rearrangement shows how the subsections of the affected chromosome are linked by junctions, duplicated are labeled in green. Inverted segments are labeled with arrows. D and E. Circos plot for RD_P26 revealing deletions (red), inversions (dark blue), and corresponding links (blue). The rearrangement and derivative chromosome are labeled for deletions (red) and inversions (blue), as well as connecting breakpoint junctions. F and G. The rearrangement of RD_P01 summarized in a Circos plot with deletions (red) and duplications (green). Duplicated segments are inserted into 21p (section 1), as well as downstream of the centromere (section 2), whereas 21q (section 3) is stitched by various inverted and duplicated segments. The sections are colored in orange, blue, and yellow. Segment N connects to a position in the centromere (cen-seq). One junction had an unmapped breakpoint (circle). H. The derivative of the complex rearrangements shows the inverted orientation of stitched segments (arrows).

Clinical analysis of RD_P505 using karyotyping, NOR staining, FISH, and array-cormparative genomic hybridization revealed an aberrant chromosome 21 (Supplemental Figure 1A-D, Supplemental Results 1). When sequenced with srGS and lrGS, the exact breakpoints of 6 duplications were determined (Table 1, Supplemental Table 2, Supplemental Figure 1E) to cover a total of 10.3 Mb (Figure 1A). The duplicated regions contained 83 protein-coding genes (Supplemental Tables 2 and 3). Furthermore, the information from srGS allowed a reconstruction of the derivative q-arm of chromosome 21 (Figure 1B and C) showing that amplified segments B and H were located in tandem. lrGS soft-clipped reads provided the evidence that segments D, J, and the inverted F were stitched together to a 3.76 Mb sequence and inserted into the region between 2.2 and 3.2 Mb (Figure 1B and C).

**Table 1:** SVs of the 3 participants provided with T2T-CHM13 breakpoint coordinates and the method that detected the breakpoints

RD_P505	Segment	Start	End	Type	Method
	B	23,765,839	24,915,464	DUP	aCGH, srGS, lrGS
	D	29,450,256	30,482,559	DUP	aCGH, srGS, lrGS
	F	30,739,588	32,660,588	DUP	aCGH, srGS, lrGS
	H	33,708,435	35,258,903	DUP	aCGH, srGS, lrGS
	J	37,439,494	38,243,038	DUP	aCGH, srGS, lrGS
	L	41,208,203	45,090,682	DUP	aCGH, srGS, lrGS, FISH

**Karyotype** seq[T2T-CHM13v2.0] 46,XY,der(21)(pter::p11.2→p11.2::q22.3→q22.3::p11.2→p12::q22.11→q22.11::q22.2→q22.2::q22.11→q22.11::p13→p13::p11.2→q21.3::q21.2→q22.13::q22.12→qter) NC_060945.1:g.[pter_(8500000_9500000)delins[7309183_(8500000_9500000)inv;41208203_45090682;3280083_7309182inv:29450256_30482559;37439494_38243038;30739588_32660588inv;pter_23765838inv];24915464_24915465ins23765839_24915464;35258903_352589034ins33708435_35258903]					

**RD_P26**	**Segment**	**Start**	**End**	**Type**	**Method**
	Tel	pter	329,999	DEL	OGM
	B	532,439	2,754,000	DEL	OGM
	C	2,754,001	3,885,184	INV	srGS∗, lrGS∗, OGM
	D	3,885,185	8,276,221	DEL	OGM∗
	F	40,385,031	41,802,370	DEL	aCGH, srGS, lrGS, OGM
	G	41,802,371	42,715,704	INV	srGS, lrGS, OGM
	H	42,715,705	45,090,682	DEL	aCGH, srGS, lrGS, OGM, FISH
					

**Karyotype** seq[T2T-CHM13v2.0] 46,XX,der(21)(::p12→p13::p13→p13::p11.2→q22.3::q22.3→q22.3::) NC_060945.1:g.[pter_(8275500_8277000)delins[2754001_3885184inv;(329000_331000)_532438]::40385031_qterdelins[41802371_42715704inv]]					
**RD_P01**	**Segment**	**Start**	**End**	**Type**	**Method**
	B	14,851,664	24,610,783	DEL	aCGH, srGS, lrGS
	D	25,290,884	25,303,024	DUP	srGS, lrGS
	F	25,713,476	25,865,801	DUP	aCGH, srGS, lrGS
	H	26,642,007	26,776,417	DUP	aCGH, srGS, lrGS
	J	26,799,626	26,934,775	DUP	srGS, lrGS
	L	27,417,474	27,809,326	DEL	aCGH, srGS, lrGS
	N	29,463,413	29,673,698	DUP	aCGH, srGS, lrGS∗
	P	31,172,424	31,179,763	DUP	srGS, lrGS
	R	31,653,158	34,549,575	DEL	aCGH, srGS, lrGS∗
	T	36,841,751	37,237,424	DEL	aCGH, srGS, lrGS
	V	42,358,829	42,440,338	DUP	srGS, lrGS
	X	43,489,001	43,505,695	DUP	srGS
	Z	44,694,487	44,862,839	DUP	aCGH, srGS, lrGS

**Karyotype** seq[T2T-CHM13v2.0] 46,XX,der(21)(pter→p13::q21.3→q21.3::q21.3→q21.3::p12→q11.2::q21.3→q21.3::q21.3→q21.3::q22.11→q22.11::p11.2→?::q21.3→q21.3::q22.11→q21.3::q22.11→q22.11::q22.3→q22.3::q22.3→q22.3::q22.13→q22.12::q22.3→q22.3::q22.2→qter) NC_060945.1:g.[2836888_3100034delins[25290884_25865801inv;24610784_25303024];14851664_37237425delins[26799626_26934775inv;26642007_26776417inv;29463413_29673698;10971588_?;25713476_27417473;27809327_31179763inv;31172424_31653157inv;44694487_44862839inv;42358829_42440338inv;34549576_36841750inv;43489001_43505695]]

FISH probes annealing to the duplicated L segments suggested a large inversion on the p-arm, which was confirmed by lrGS and NOR staining (Supplemental Figure 1F, Supplemental Results 2).

Sequencing the parents with liGS enabled the phasing of SVs and SNVs detected in the participant (Supplemental Figure 1G and H). The result is a significantly increased ratio for paternal SNVs of around 0.6 compared with that of maternally phased SNVs of 0.3 in duplicated segments compared with unaffected regions, indicating that the rearrangement occurred in the paternal allele.

In total, 7 of 9 junctions were resolved at the nucleotide level in the derivative chromosome. A detailed analysis of those junctions revealed microhomology in 6 of 7 junctions (size 1-79 nucleotides). The longest microhomology was part of an *Alu* element located on both sides of the rearrangement (in junction A2D). One junction showed a 4-nucleotides-long nontemplated insertion without microhomology (A2D) (Supplemental Figure 2, Supplemental Table 2).

### RD_P26: A ring chromosome

RD_P26 was previously described with a ring chromosome 21 containing 2 terminal deletions on 21q detected by FISH and CMA.^5^ srGS, lrGS, and OGM confirmed and pinpointed the exact location of the 2 deletions in 21q22.3 (Figure 1D, Table 1) leading to the loss of 37 protein-coding genes (Supplemental Tables 3 and 4). Moreover, the in-between segment was inverted.

Additionally, lrGS and OGM uncovered reads originating from segment G placed in inverted read orientation on 21p in segment C (Figure 1E, Supplemental Figure 3A and B). Soft-clipped lrGS reads bridged the q- and p-arm connecting to 21p12 at 3.1 to 5.6 Mb, which is known as the NOR-region or ribosomal DNA satellite region. The remaining p-arm was reconstructed using the OGM data (Supplemental Results 3).

The junctions EG and GC were resolved at the nucleotide level (Supplemental Figure 4A and B), presenting a complex local microarchitecture and blunt ends, respectively (Supplemental Results 4). The presence of *Alu* element sequences in junction EG suggests an initial 21qter inversion event that renders the q-arm prone to further rearrangements leading to the ring formation (Supplemental Table 5).

Next, phasing blocks and SNVs from liGS delineated the hemizygous segments F and H to the paternal allele, concluding that the 2 deletions happened on the maternal allele (Supplemental Figure 4C). No SVs were found in the parents on 21q23, labeling the rearrangement in the participant including the ring chromosome as *de novo*.

### RD_P01: Chromoanasynthesis with deletions and duplications

RD_P01 was sequenced with srGS and lrGS, showing a complex rearrangement containing 13 SVs and 26 breakpoints (Figure 1F). The additional gain of resolution compared with previous publications^6^^,^^13^ is 1 additional breakpoint and 3 more solved junctions (Supplemental Results 5). The data confirmed the presence of 4 deleted segments (B, L, R, and T) totaling 13.4 Mb, affecting 31 genes and 9 duplicated segments (D, F, H, J, N, P, V, X, and Z) totaling 1.9 Mb, duplicating 13 genes (Supplemental Figure 5A, Table 1). A detailed list of disrupted genes involved repeats and coordinates can be found in Supplemental Tables 3 and 6.

The lrGS data increased spatial resolution for the 3 derivative sections by phasing the SV breakpoints in cis: F-D, J-N, and F-A2 (Figure 1G, Supplemental Figure 5B and C, Supplemental Results 6).

Next, the aberrant chromosome 21 was analyzed for its inheritance. The SNVs in diploid segments showed a balanced ratio of 0.5, whereas the majority of phased SNVs in deleted regions were found in the mother and therefore phased the rearrangement origin to the paternal allele (Supplemental Figure 5D).

The final derivative chromosome (Figure 1H) contains 15 junctions (Supplemental Figures 6 and 7) that showed a combination of 3 insertions of 26 to 94 nucleotides (junctions 4, 8, and 12), 2 sequence similarities around breakpoints (junctions 1 and 9), 13 microhomology of 1 to 7 nucleotides (junctions 1-4, 6-7, and 9-15), 2 nontemplated insertions of 4 to 9 nucleotides (junctions 6 and 9), 1 short inversion (junctions 14), and 1 simple repeat (junction 8).

## Discussion

Here, we report 3 research participants with complex chromosome 21 rearrangements. Through the integration of srGS and lrGS technologies combined and traditional cytogenetic methods, we successfully reconstructed the rearrangements uncovering a high level of previously unknown complexity. Furthermore, by aligning the genome data to T2T-CHM13, we demonstrate the involvement of breakpoints located between 21p13 and 21p12 in all 3 derivative chromosomes.

The short arm of chromosome 21 has a high number of satellite sequences, including type A, B, and C.^25^ These satellite sequences are not unique to chromosome 21 but are shared with the other acrocentric chromosomes—Chr13, Chr14, Chr15, and Chr22—and recombination is common between those repetitive sequences as observed in Robertsonian translocations.^14^^,^^26^ All 3 cases studied here harbor at least 1 rearranged genomic segment from 21q that was inserted or copied to 21p12. The location is mapped between 2.8 to 3.2 Mb, suggesting a hot spot for genomic rearrangements in the vicinity of NOR. This repetitive region is traditionally difficult to analyze because of low sequence complexity, megabase-scale repeats, and high degree of similarity with other acrocentric chromosomes. First, the length and number of repeats in and around NOR vary greatly between participants.^26^^,^^27^ Second, the NOR of all 5 acrocentric chromosomes are not always present or sporadically inactive independent of whether it is a homologous or heterologous chromosome.^14^^,^^27^ Additional studies of SVs involving chromosome 21p are needed to confirm whether or not 21p12 is a genomic hotspot for chromosomal rearrangements.

Historically, the ability to detect rearrangements in acrocentric p-arms has been limited by the coverage in the available reference genomes. Of note, publications from before the first human reference genome was released, have used cytogenetic methodology to query those genomic regions with low resolution. In such studies, gross chromosomal rearrangements will be identified but not resolved to the nucleotide level. Previous work showed that the p-arm is involved in case RD_P01^6^; however, only 16 of 26 breakpoints were detected, and the suggested derivative chromosome 21 lacked information on 5 (out of 9) duplicated segments of which 3 are inserted into the p-arm. When the same structure was analyzed by srGS and GRCh37, the derivative chromosome structure was much more complete,^13^ but as expected, it still lacked specific information for the p-arm. GRCh37 is a well-annotated established reference with good-quality sequencing information in unique regions dense with protein-coding genes, such as 21q, although devoid of information in the 21p region. The GRCh38 reference has better coverage in coding and noncoding regions, whereas low-complexity regions consisting of low-copy repeats, centromeres, telomeres, and satellites remain a challenge.^28^ In fact, for all 3 CGRs reported here, the 21p rearrangements could not be resolved with GRCh38.

The T2T-CHM13 reference performs better for specific cases, such as those detailed here, because it contains the full acrocentric p-arm and centromere sequences.^12^ Although this reference allows us to study the detailed architecture of CGRs involving acrocentric chromosomes, the analysis is hampered by the lack of populational SV/haplotype databases. With new possibilities to study acrocentric p-arms, it becomes apparent that the satellite regions are variable among the population. Such variability may amount to several megabases of sequence and limit our ability to differentiate the disease-causing SVs from background variation. Better-quality *de* *novo* assembled parental genomes might help overcome this issue, but for the cases studied here, the parental DNA was only analyzed by liGS, and the contigs covering 21p were too short.

**Figure 2:**
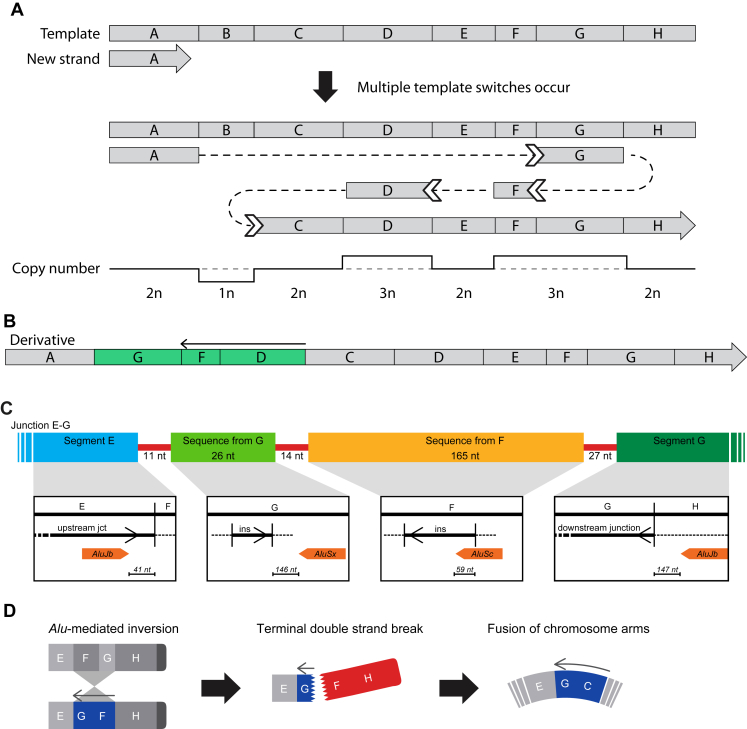
**Mechanisms of the rearrangement in the three participants.** A and B. RD_P505 and RD_P01: Exemplified view of chromoanasynthesis showing multiple template switches and reannealing (block arrow) leading to duplicated segments (green) and inversions in the derivative chromosome. C. Overview of the junction EG in RD_P26 illustrating inserted segments (light green and yellow), flanking segments E (blue), G (dark green), and *Alu* elements (orange) that may have facilitated the *Alu*-mediated inversion. D. Proposed 3-step mechanism for RD_P26 that forms the ring chromosome: An *Alu*-*Alu*-mediated recombination generates a terminal inversion (blue) followed by a terminal deletion rescued by the ring formation to stabilize broken ends.

The 3 CGRs resolved here display highly different characteristics, suggesting that they were formed through different mechanisms. Both RD_P505 and RD_P01 are likely to have occurred through the replicative mechanism chromoanasynthesis^29^ (Figure 2A and B) with at least 9 and 13 template switches, respectively.

In RD_P505, the described derivative chromosome structure with 6 copy-number gains and the microarchitecture of the 7 junctions show microhomology, *Alu*-*Alu* fusion, and a small 3 nt templated insertion (Supplemental Figure 2).^30^^,^^31^ In RD_P01, the 26 breakpoints result in 4 deletions and 9 duplications sized from <10 kb to 10 Mb. Microhomology is present in 13 of 15 junctions, and some, but not all, duplicated segments are inverted. Considering that segment A on the q-arm is also connected to the centromere, it is possible that the derivative A(q11.2)-JHN-cen is a pericentric inversion. In such a case, we would expect an additional breakpoint in 21q11.1, which might then be hidden in the centromere.

In RD_P26, the complex junction EG containing 2 insertions had 6 breakpoints located within or in proximity (41, 146, 59, and 147 nucleotides) of *Alu* elements (Figure 2C, Supplemental Table 5). The sequences at 21q22.3 in breakpoint G are highly similar to parts of 21p12. In GRCh37 and GRCh38, the site is annotated as a segmental duplication, but this is not yet the case in T2T-CHM13. The sequences, however, still share identity, which we believe to have facilitated the pq-arm bridge. Therefore, we propose that the ring chromosome formed through a 3-step event starting with an *Alu*-mediated rearrangement, and an inversion of a segment (F+G) as initiator followed the loss of terminal ends. The formation of the ring chromosome as the final step stabilized the broken ends. The sequences at the breakpoints share a high degree of similarity, which facilitates the chromosomal repair (junction GC) (Figure 2D).

The clinical phenotypes differed between the 3 cases with some common features, such as variable developmental delay. The phenotypic presentation of case RD_P505 is extremely similar to DS. Interestingly, the highly restricted DS critical region,^32^^,^^33^ a 34 kb segment located at 21:36,312,745-36,346,647, has a normal diploid copy number in RD_P505 (segment I), and the closest duplicated regions are located 1 Mb upstream and downstream (segment H and J, 1.55 and 0.8 Mb, respectively) of highly restricted DS critical region. The less restrictive larger DS critical region at 21q22.13-q22.2 has been linked to a milder DS phenotype.^34^ Both segments H and J overlap with that region and likely contribute to the DS symptoms in RD_P505. It is, however, not possible to fully determine the impact of the other duplications (totaling 8 Mb of sequence and 69 genes) (Supplemental Tables 2 and 3). Similarly, for RD_P26 and RD_P01, the exact contributions of the 37 deleted genes (RD_P26, Supplemental Table 4) and the 31 deleted and 13 duplicated genes (RD_P01, Supplemental Table 6) remains to be determined. For RD_P26, it was previously shown with the RNA sequencing of patient-derived NESCs that multiple genes had aberrant expression levels likely involved in the participant’s phenotype.^5^ Another possibility for the symptoms in this ring chromosome case (RD_P26) is that the clinical manifestations, such as growth failure and intellectual disability, are due to ring chromosome replication instability during mitosis and associated apoptosis.^35^^,^^36^ This is credited to the fact that ring chromosomes *in vivo* are often mosaic after mitosis when they undergo both breakages and fusions.

With the advancements in genomic sequencing methodologies, our capacity to identify and understand complex genomic events has expanded. A growing number of reports on clinically relevant CGR with unexpected complexity are published in scientific literature.^37^^,^^38^ Contrary to the initial perception of CGR as rare events, they are more common, partly because of the enhanced capabilities of 3rd generation genomic sequencing technologies.^7^ These technologies generate longer reads, enabling the detection of rearrangements *in cis*, and improve the resolution of intricate genomic regions, such as repeat elements, segmental duplications, centromeres, and telomeres.

When analyzed cases are published, the methods align with the state of the art at the time of publication. This means that early SV callers, in the context of CGR, might be inferior to updated and refined methods. Specifically, early SV callers need to handle long-read sequencing data, which differ from short reads in terms of length, read depth, and quality. Over the past year, SV callers have undergone updates to provide finer call results, increasing sensitivity and specificity. Consequently, reanalyzing older cases with the new pipelines enhances the diagnostic yield without the need for resequencing. This iterative process ensures that reanalysis benefits from improved methodologies and contributes to a more comprehensive understanding of CGR.

In conclusion, we demonstrate that the increasing quality of detection methods and reference genomes makes it possible to resolve complex genetic aberrations located even within the p-arms of acrocentric chromosomes. Furthermore, the p-arm of chromosome 21 might be prone to chromosomal rearrangements especially the region around the ribosomal DNA region.

## Data Availability

The genomics data supporting the conclusions of this study are available upon request. Please contact the corresponding author.

## Conflict of Interest

Anna Lindstrand has received honoraria from Illumina and Pacific Biosciences. All other authors declare no conflicts of interest.

## Additional Information

Supplementary Table 1

Supplementary Result
