# Supplementary material for: A combination of long- and short-read genomics reveals frequent p-arm breakpoints within chromosome 21 complex genomic rearrangements
Source: Genet Med Open. 2024 Jun 28;2:101863. doi: 10.1016/j.gimo.2024.101863 (PMC11613786; doi:10.1016/j.gimo.2024.101863)
Supplement: Supplementary Table 1 [file mmc1.docx]

## Supplementary Results:

**Results S1: RD_P505 - Cytogenetic results**

RD_P505 presented with moderate developmental delay, microcephaly and displayed dysmorphic features similar to Down syndrome. Clinical analysis with karyotyping, FISH and CMA revealed an aberrant chromosome 21 with a total of five duplications on 21q. First, karyotyping showed the presence of extra material of unknown origin on the p-arm in the size of several megabases as well as an increase of chromatin flanking 21q22.12 suggesting at least two duplications on the q-arm (Figure S1A). Subsequently, FISH revealed that the duplicated material from 21q was inserted in the terminal p-arm region and showed an extended region between *RUNX1* in 21q22.12 and 21qter (Figure S1C). Finally, CMA confirmed the presence of two duplications in 21q22.12 and q22.3 of 1.55 Mb and 3.88 Mb (Figure S1D) and detected three additional duplications at q21.3, q22.11 and q22.2 sized 1.15 Mb, 2.95 Mb and 0.8 Mb, respectively.

**Results S2: RD_P505 - p-arm inversion with detected with NOR and FISH**

To resolve the remaining derivative 21p, the integration of multiple data sources was necessary. In this participant, metaphase chromosomes were stained for NOR, a region with active ribosomal RNA genes on the p-arm of acrocentric chromosomes. The NOR-staining showed no active NOR on the wild-type allele while the derivative chromosome showed two strong signals on both distal chromosomal arms (21p12 and 21q22.3) and a faint signal between the centromere and 21pter located adjacent to 21p12 (Figure S1B). The FISH data, with probes annealing to segment L, showed that L was present in two locations (21pter and 21qter) and several lrGS split reads map to that same area on 21p (between 7.3 – 7.4 Mb) orientated towards the centromere (Figure S1F). The position at 21p12 is where the DJF segment was then inserted separating the NOR signal. In combination with the FISH result, the derivative 21p then contained a large inversion spanning A1-A3 that brings segment L distal while NOR is relocated towards the centromere. We could however not find evidence in any GS dataset for an inserted 21p12 on the q-arm which the third NOR signal suggests.

**Results S3: RD_P26 - OGM for the p-arm reconstruction**

The p-arm of the derivative chromosome could not be resolved by lrGS due to megabase-scaled repeats. OGM was therefore used which showed a partially unaligned assembly in segment G (Figure S3A) with a distinct “barcode” pattern which was manually aligned to the NOR at 21p12 (Figure S3B). We utilized the copy number diagram which showed a diploid genome between 3.2 and 3.7 Mb to narrow down the breakpoint. Segment C starts around 2.75 Mb which is visualized by a diploid copy number and aligned assembly, the latter also showed two unaligned molecules which were linked to the same region (Figure S3C). The unaligned tail was manually mapped closer to the subtelomeric region after inverting the assembly (Figure S3D), creating segment A between 0.3 Mb and 0.55 Mb. We verified the OGM assembly and found at least one molecule spanning the G-C and C-A junctions. We were not able to find the breakpoints in the lrGS data to confirm the CA junction. However, due to the inverted assembly reaching segment A, the derivative chromosome is now directed towards the centromere as would be expected in a ring chromosome.

**Results S4: RD_P26 - Break point junction analysis**

In EG a complex local micro-architecture was found with a 243 nucleotides insertion that contains three non-templated segments, 26 nucleotides from segment G (21:42,401,952-42,401,977), and 165 nucleotides from segment F (21: 41,732,407-41,732,587) (Figure S4A). The junction GC revealed blunt ends (Figure S4B).

The breakpoints on the p-arm were located in the repetitive NOR at 21p12 and could not be resolved at the nucleotide level since they were only captured by OGM (corresponding to junctions CA and AE).

**Results S5: RD_P01 - Additional gain compared to previous publications**

RD_P01 was first analyzed in 2010 (Patient 1), with karyotyping and CMA unraveling four deletions and four duplications on chromosome 21.^6^ Next, in 2018, srGS analysis showed additional variation and higher resolution detecting a total of 25 breakpoints, four deletions and nine duplications of which six were inverted (Participant P1426_301).^13^ However, the CGR could not be fully resolved at the time with four breakpoints remaining unmapped due to the high repetitive sequence content. In this study, segment labels from the previous srGS publication (A-Z) were kept and one label (A2) was added at the end of the derivative (Figure 1F). However, in the current study, the T2T-CHM13 reference was used and therefore, coordinates will differ compared to previous published work in GRCh37/hg19.

**Results S6: RD_P01 - detailed description for the three rearrangement sections**

The region of D was fully covered by one lrGS read revealing that segments C and duplicated D were directly linked to each other and inverted relative to the DE connection (Figure S5B). Moreover, multiple lrGS reads showed that the section FEDCinvDinv was inserted into 21p13, directly upstream of the NOR (21:3,100,000 – 5,600,000).

In the J-N section, the duplicated segments J and H are within the same phase block (Figure S5C) located in inverted orientation on 21q downstream of the centromere (21:14,900,000). The breakpoint downstream of N was only detected by srGS and liGS and for both, the informative reads lead into the centromeric sequences (alpha-satellite sequence) and cannot be precisely located.

Segments F-A2 are not completely resolved. lrGS supports a junction connecting the start of F with 21:44,520,000 between segments X and Y where four similar simple repeats are located (GCGCCCTCCCCCCCCG, GCCCTCCCCCCAACAG, CCCTTCCCCCTCCCCG, CGCCCTCCCCCCAACA) (Figure S6). The two informative reads from lrGS cover the whole sequence including the simple repeats and the soft-clipped sequence matches uniquely to this site providing evidence for a true hit. The repetitive genomic region was too complex for srGS visible by a fluctuating read depth. However, the read depth in the lrGS data does not support the presence of a duplication at 21:44,520,000 and we also do not detect a breakpoint upstream that would complete the derivative Chr. Instead, srGS and liGS data suggested a link between segment S to upstream of X, and segment U to downstream of X. Even though neither connection SX nor UX are supported by lrGS, this option is more likely since it results in a complete derivative chromosome (Figure 1F).

The main advantage, beyond the increased p-arm resolution, of lrGS and T2T-CHM13 compared to the previously published srGS/GRCh37 analysis,^13^ was the more complete aligning across merged duplications such as segments FEDCD and JHN. The segment N in AJHN contains a link to the centromere, but the exact location is yet unknown.
