# Supplementary material for: A combination of long- and short-read genomics reveals frequent p-arm breakpoints within chromosome 21 complex genomic rearrangements
Source: Genet Med Open. 2024 Jun 28;2:101863. doi: 10.1016/j.gimo.2024.101863 (PMC11613786; doi:10.1016/j.gimo.2024.101863)
Supplement: Supplementary Result [file mmc2.pdf]

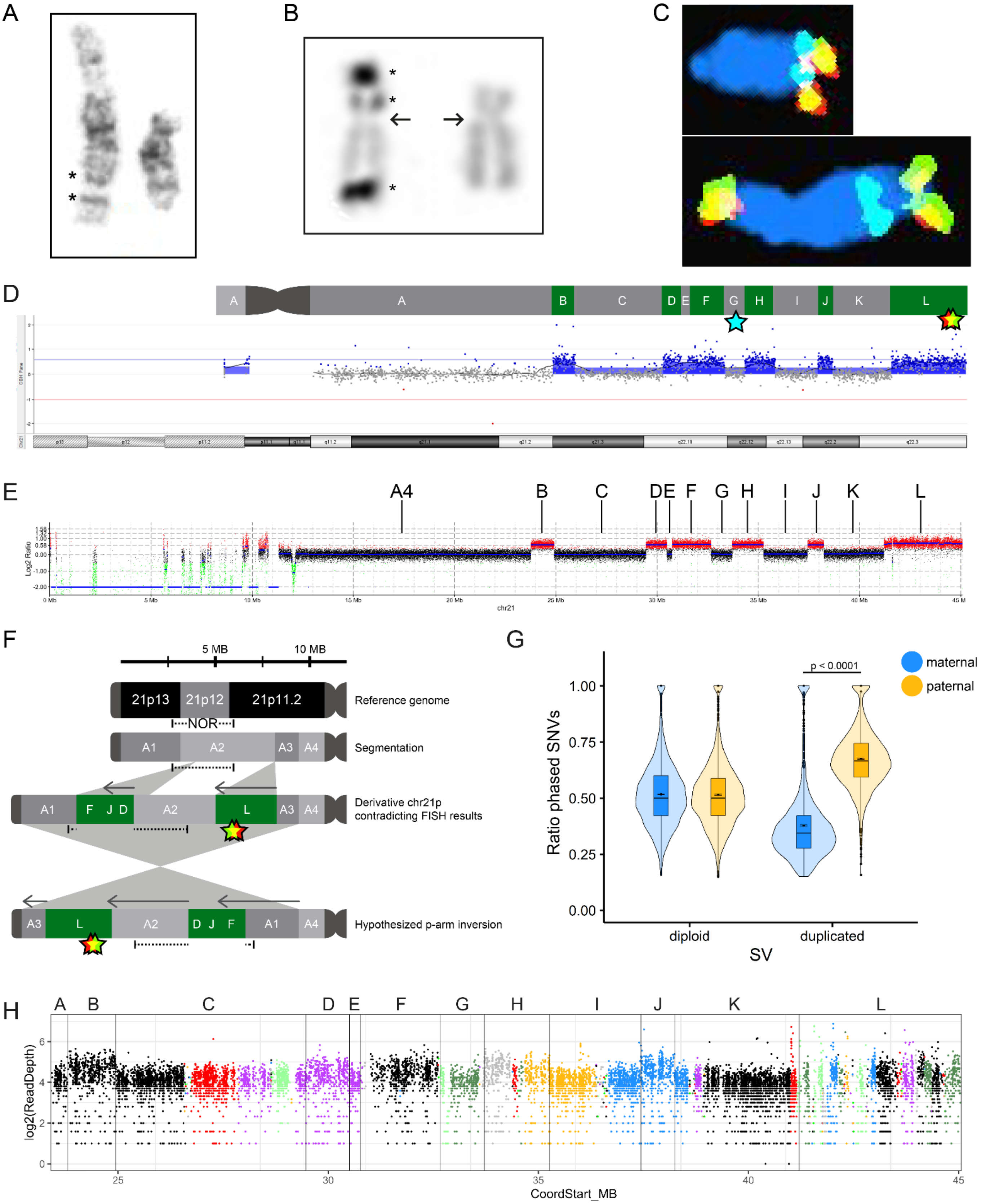

Figure S1: Genomic findings for RD_P505: (A) The chromosome 21 taken from the karyogram revealed an elongated chromosome 21 with additional material on 21q (asterisks)

when compared to the healthy allele. (B) AgNOR staining labelling active ribosomal DNA transcription sites (asterisks) in affected allele (left) compared to unaffected chromosome 21 (right). The centromeres are marked with arrows. (C) Fluorescence *in situ* hybridization (FISH) probes annealing to *RUNX1* (cyan) and subtelomeric regions on 21q (green, red) showed duplicated genomic material on both chromosomal arms (left) compared to the unaffected chromosome (right). (D) CMA reveals six duplications (green) and the locations for the FISH probes (cyan, light green, red). (E) Copy number plots visualizing read depth of srGS (top) and lrGS (bottom) labelled for all segments and colored for copy number gains (red) and copy number losses (green). (F) The 21p derivative shows whole segment inversion that explains distal FISH signals (green, red) and the divided NOR staining (dotted line). (G) Trio analysis revealed a 2:1 ratio of phased SNVs in the duplications between the maternal (blue) and paternal allele (yellow). (H) liGS data from 21q shows phasing blocks (various colors) demonstrating which segments were partially or fully phased.

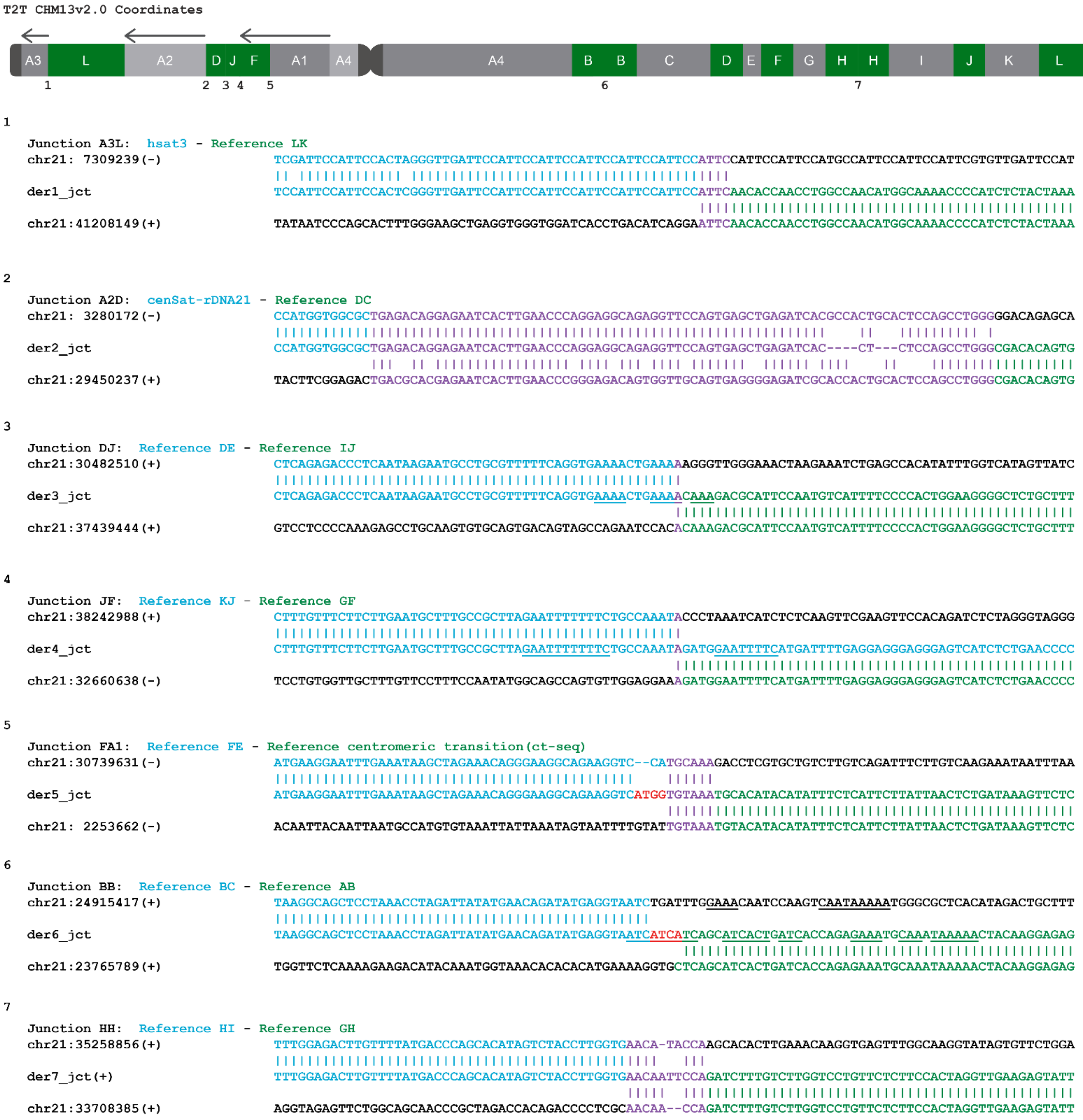

Figure S2: Breakpoint junction analysis of RD_P505: Seven breakpoint junctions have been resolved based on the derivative chromosome. The breakpoints contain microhomology (purple), non-templated insertions (red) and other sequence features like sequence similarity around the breakpoint (underscore).

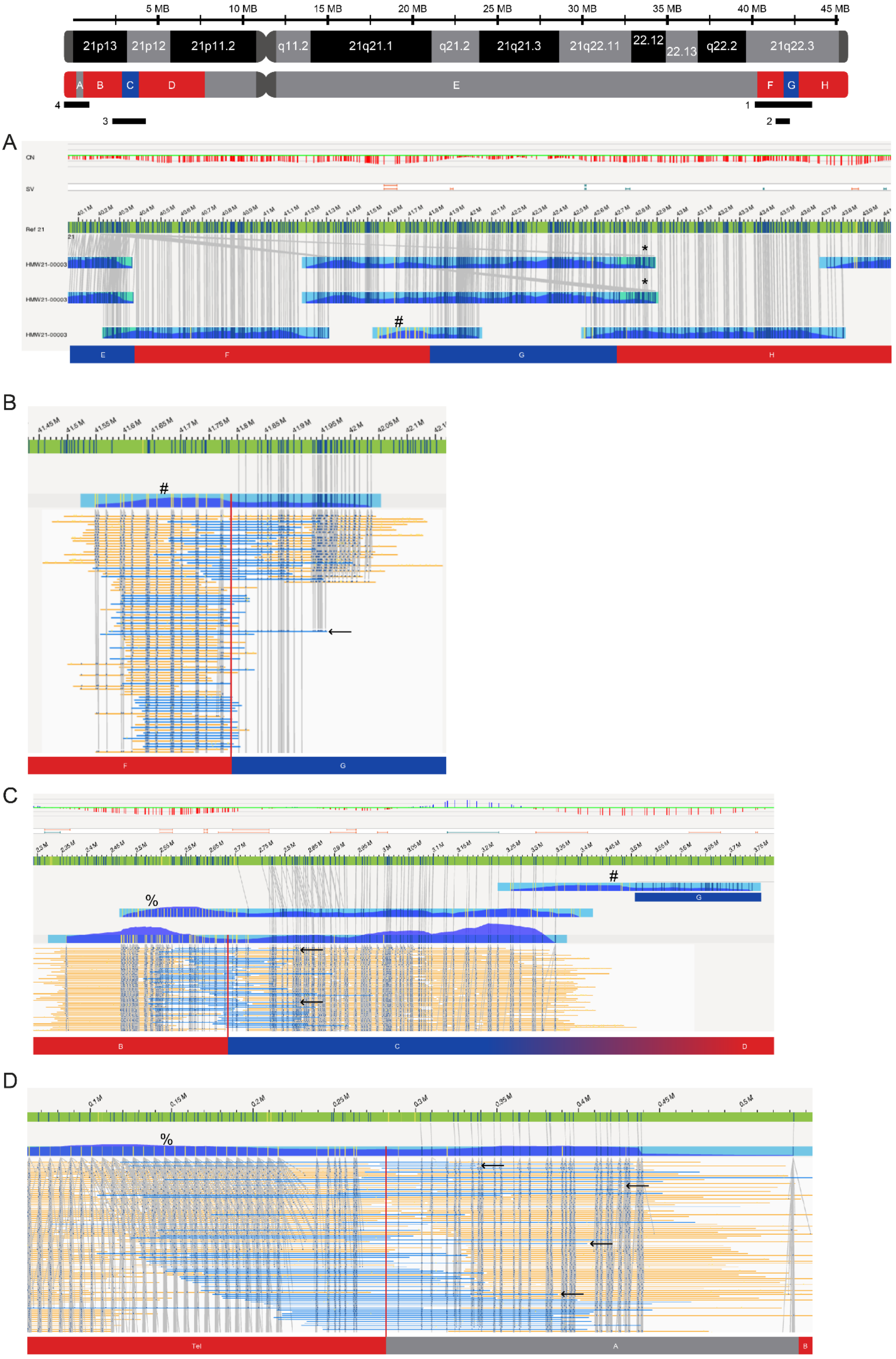

(figure legend on the next page)

Figure S3: Optical genome mapping data for RD_P26 rearrangement. The data was visualized in Bionano Access showing the copy number (CN), reference genome T2T-CHM13 (green) with label pattern (blue barcode), assembled molecules (light blue) with internal coverage (dark blue) as well as label for SVs E-H in RD_P26. (A) Junctions on 21q showing the two deletions (F, H) and inversion (G) with misaligned parts (turquoise, asterisks) that contain a sequence of 21p (pound). (B) Quality control of F-G junction showing single molecules (yellow) bridging the full breakpoint (red line) and thus verifying the junction (arrow). (C) Overview of the 21p region BCD showing aligned molecules from 21q (pound, G), two haplotypes with different unaligned molecule extensions (%). The breakpoints were verified by bridging molecules (arrows), The copy number decreased up and downstream of segment C (segment B and D). (D) Quality of the junction (red) starting in A turning back to the centromere, labelled for bridging molecules (arrows).

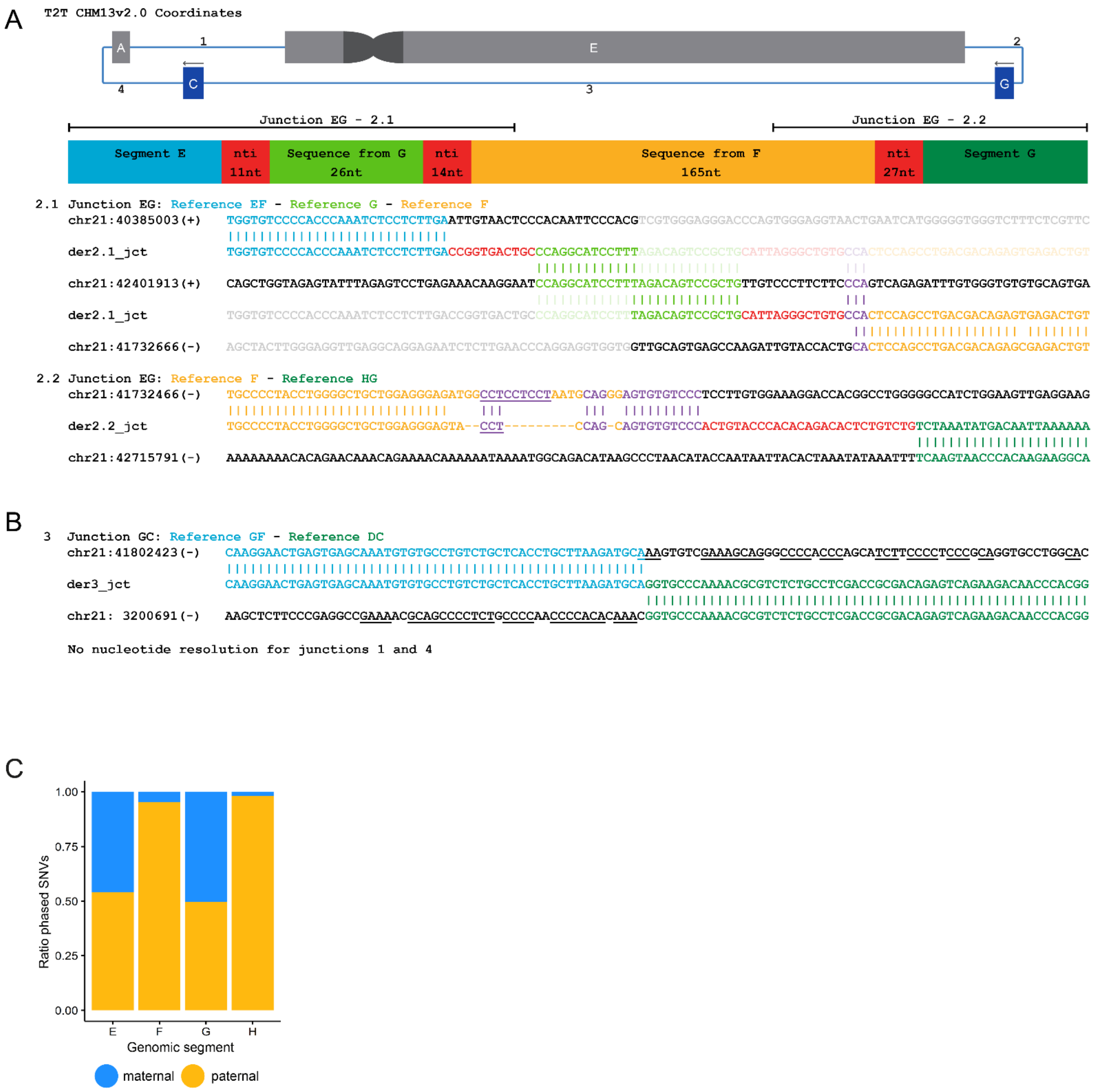

Figure S4: Breakpoint junction analysis in RD_P26 and haplotyping. (A, B) Two of four breakpoint junctions have been resolved on the nucleotide level showing non-templated insertions (red) and microhomology (purple). Junction EG carried additional insertions from segment G (light green) and F (orange) that were flanked by non-templated insertions (nti). Breakpoint junction GC contained blunt ends. Both junctions carried similar sequence features around the breakpoints (underscore). (C) Trio analysis of SNVs reveals that the remaining SNVs present in the deleted segments are linked to the father, showing that the ring chromosome is from the maternal allele.

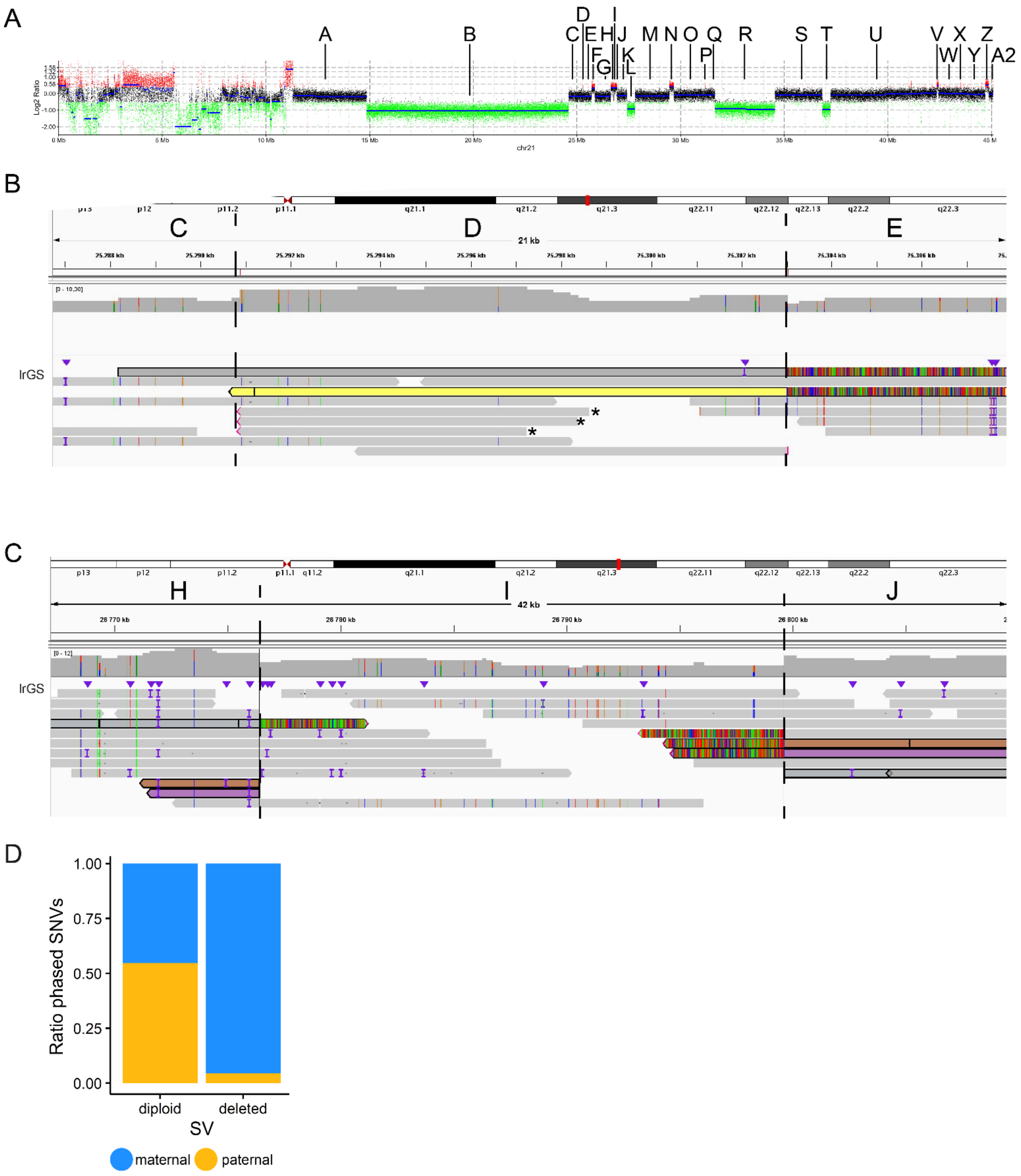

Figure S5: Short read and long read genome sequencing analysis of RD_P01. (A) Copy number plots visualizing read depth of short read genome sequencing (srGS) labelled for all segments and colored for copy number gains (red) and copy number losses (green). (B) Aligned lrGS reads fully spanning segment D (outlined in grey and yellow) connecting C to D

tail to head and to 21p downstream of D. Hard clipped reads in D connect to C head to tail (asterisks). (C) Excerpt from segments H-I-J show lrGS reads that connect the duplicated segments J and H to each other. (D) The trio analysis of SNVs revealed that the paternal allele carries the rearrangement while only maternal SNVs remain in regions for deletions.

T2T CHM13v2.0 Coordinates

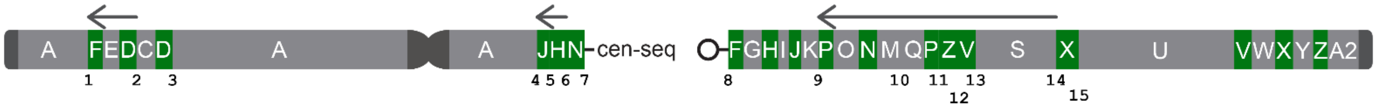


```
1  Junction AF: Reference centromeric transition(ct-seq) - Reference GF
   chr21: 2836833(+)   TCCCTGTTGTCATCCTTATCATGGATTAAAAGTCACCTCAGTGAGGCCTTAGGTCCTCCCATGCAATAATTTTCCAGCTTTTCTTCTCAACATTCCACTTTATATTATAG
   der1_jct            TCCCTGTTGTCATCCTTATCATGGATTAAAAGTCACCTCAGTGAGGCCTTTGATCATAATTTTATATGTCAATTAAAAATAAATTTAAAAAATAGGACAGGTGTATTTTT
   chr21:25865855(-)   TCATTCTACAATGTGTATGTACTTCAAAACATTATGTTATGCATGATAAACACACATAATTTTATATGTCAATTAAAAATAAATTTAAAAAATAGGACAGGTGTATTTTT

2  Junction DC: Reference DC - Reference BC
   chr21:25290939(-)   GAGACGGGGTTTCACTATGTTGGCCAGACTGGTCTCACACTCCTGACCTTGTGATCCACCCTCCTCGGCCCCCTAAAGTGCTGGGATTACAGGCATGAGGCACCACGCCC
   der2_jct            GAGACGCGGTTTCACTATGTTGGCCAGACTGGTCTCACACTCCTGACCTTGTGATCATAGTTGTCCTGGTGTCTTTAAAGAAAATAATGGCTATAAAGAAATCCTTTACA
   chr21:24610729(+)   TCTTGCAGACAGTGGTGCCCAATATATGTTTGCTAAATAAAAGAACAAATGATGGCATAGTTGTCCTGGTGTCTTTAAAGAAAATAATGGCTATAAAGAAATCCTTTACA

3  Junction DA: Reference DE - Reference centromeric transition(ct-seq)
   chr21:25302968(+)   ATCTAGGACAGTAGTTTGAGCAATTACAAACTCTAAAAAGTAGACAGCAAATCATCCTAGTGGTGTTTTTAACAAAGCTTTCTAGAATCATACACAAACCACTAAAACAA
   der3_jct            ATCTAGGACAGTAGTTTGAGCAATTACAAACTCTAAAAAGTAGACAGCAAATCATCCCACCTTGGTCTTCAGAAGTCCTGGGATTACAGGCTTGAGCCACTGTGCTCACT
   chr21: 3100088(-)   TCTCTCTCGATGTGTTGCCCAGGTTAGTCTCAAACTCCTGTCCTCTATCCAGCCTCCCACCTTGGTCTTCAGAAGTCCTGGGATTACAGGCTTGAGCCACTGTGCTCACT

4  Junction AJ: Reference AB - 500bp upstream BP-A - Reference KJ
   chr21:14851651(+)   ATAGACAAATCTGGTTTCTTTCTTAGAGAAACTTAGACTGTAATCATTATAATGATATAGATGGAAGTTAATTCATTGATGTAAGTGGATTAATTCAGCAGTATGTATAT
   der4_jct            ATAGACAAATCTGTCCCTCCTCCTCCTGCCCTCTACATTTCAAACACATCGTATTGCTATTTCTCAAGTGCCATTATTATTTGTTATGATGCTCGGATTTTGCTTTTTGC
   chr21:14851168(-)   CCTACTTATCTTGTCCCTCCTCCTCCTGCCCTCTACATTTCAAACACATCGTATTGCTATTTCTCAAGTGCCATTATTATTTGTTATGATGCTCGGAGCTCCCTTATCCA
   der1_jct            ATAGACAAATCTGTCCCTCCTCCTCCTGCCCTCTACATTTCAAACACATCGTATTGCTATTTCTCAAGTGCCATTATTATTTGTTATGATGCTCGGATTTTGCTTTTTGC
   chr21:26934874(-)   TACCTACTTCCATGACCATCCAGAACCAAATTTTGTTTGTATGACAAAAAGAACATTGGGCTTTTTTTTTTGACTAATCAATCACCAATAACATTCTGGTTGCTTTTTGC

5  Junction JH: Reference JI - Reference IH
   chr21:26799669(-)   GGCTGTCTTTGCTCATTATCCTGAGCATGGCTGACTCATGGTGTTCCATGCTTTGTTCCCTGGCTTTTGTTCCTTGGCCTGTGACTCACCTCCTCCATGCTTTCTCCACC
   der5_jct            GGCTGTCTTTGCTCATTATCCTGAGCATGGCTGACTCATGGTGTGTGTATCTTGAACATGTGATAAAGTCTTTAGTAGAAAGAATCAGTAGCACAATTCCTCAACATGGC
   chr21:26776484(-)   AATGGTAAGAATCTATCCGTATTAGTCAGCATGAAGAGCAGGTTACTTAATTCAAATGCCCCAAATGGTCTTTAGTAGAAAGAATCAGTAGCACAATTCCTCAACATGGC

6  Junction HN: Reference HG - Reference MN
   chr21:26642054(-)   TACTAAATTTCTCAAAGCTGTTGAAATAATAATCTGTAAAGGGAAATCACATGTAGCTTTGCCTGAACTCTTTTCAACATCTGTGTTGTGTGGAATAGTAAAAAACACTT
   der6_jct            TACTAAATTTCTCAAAGCTGTTGAAATAATAATCTGTAAAGGGAAATCTTTATCACATTTCAGCACCCACATAATAAGCACAGTACCCAATAGGTAGATTTTTAA-TTAT
   chr21:29463357(+)   TGTCCAGGTTTGTTACATACATATATTGAGTGGCATGGGGGTTTGGTGTACAGATTATTTCAGCACCCACATAATAAGCACAGTACCCAATAGGTAGATTTTTAAATTAT

7  Junction N-cen: Reference NO - Reference centromer(cen-seq)
   chr21:29673643(+)   GTGTCCTCACAATCCAGGCAGAGCACAGGATGGTGGCACACAACGAGACAGAGGAGCACACTTGAAGGGGTGCCTGCAGGCCAAATCAGGGACAATTTGAGTACCAAAAT
   der7_jct            GTGTCCTCACAATCCAGGCAGAGCACAGGATGGTGGCACACAACGAGACAGAGGAGTGTTTCAAAACTGCTCTATCAATAGAAATGTTCAACTCCTTTGGCTGGGTACAC
   chr21:10971535(+)   TCCAACGAAATCCTCCAAGCTATCCAAATATCCACCTGCATTTTCCACAACAAGAGTGTTTCAAAACTGCTCTATCAATAGAAATGTTCAACTCCTTTGGCTGGGTACAC

8  Junction 44.52-F: Reference 44.52-Y - inserted repeat pattern
   chr21:44524845(+)   GGGCGGCCCCACCGCGCCCTCCCCACCCCGTGCCCTCCCCCCAACAGCGCCCTTCCCCCTCCCCGCCCTCCCCCCAACAACTGCGCC-TCCCCTGCCCCTCCC
   der8_jct            GGGCGGCCCCACCGCGCCCTCCCCACCCCGTGCCCTCCCCCCAACAGCGCCCTTCCCCCTCCCCGCCCTCCCCCCAACAG2x[CGCCCTCCCCCCAACAACTG
                                                        a                    b                   a                         c

   Junction 44.52-F:   inserted repeat pattern at XY bp - Junction EF
   der8_jct            CGCCCTCCCCTTCCCCCTCCCCGCCCTCCCCCCAACAG]CGCCCTCCCCCCAACAAAATCTGGTTTTATGGCAAGCTAATAACAATTACCAGTGAGGTAGGTGTTAATGT
                                 b                        a                    c
   chr21:25713319(+)   TCAATGACAGAAAATGAAACTTTGAGAATTTATGGAATCTAGTTTTACAGTTTATATATGGAATATAGTATGTATTTAATGATATTAATATTTTTAGGCATTGTAGAAAT

9  Junction KP: Reference KL - Reference QP
   chr21:27417418(+)   AAAATTCAGGCACTCAGCAGATTACGGCAAAACCAAAAAGTAATCAACTTGAATCTTTCCATTTATGTTTGATTTTTCATTTTCTCATGACAGATTTTGAAATGGAATCA
   der9_jct            AAAATTCAGGCACTCAGCAGATTACGGCAAAACCAAAAAGTAATCAACTTGAATCTGAGA-TTTTGTTGTCGCTTCAGAATTTCTGCTGCATGGCAGGAACTGTGACGGT
   chr21:31179833(-)   ACTCTCAGATGTAAGTAATTTCTGTGTTCTTCTTTTTTAAAAAGCATGTCTAGTCTGACAGTTTT-TCAGAGCTTCAGAATTTCTGCTGCATGGCAGGAACTGTGACGGT
```

(figure legend on the next page)

Figure S6: Breakpoint junction analysis in RD_P01 junctions 1-9. RD_P01 carries 15 resolved breakpoint junctions that contain microhomology (purple), non-templated insertions (red) and small insertions (yellow, orange). Aligned nucleotides are shown with vertical lines.

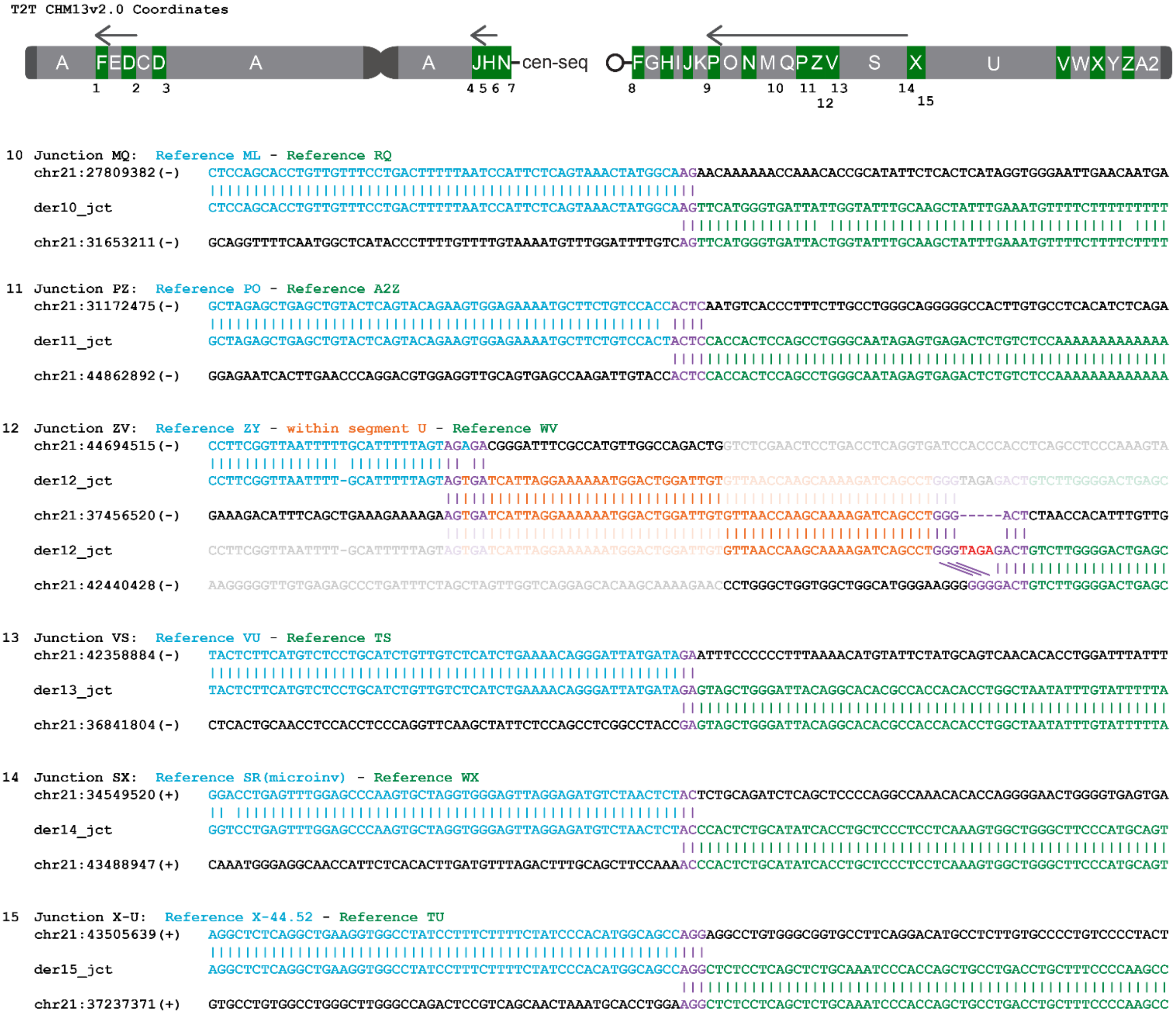

Figure S7: Breakpoint junction analysis in RD_P01 junctions 10-15. Nucleotide resolution for the breakpoint junctions shows microhomology at the breakpoints (purple), non-templated insertions (red) and small insertions (orange).
